# Beyond EUV lithography: a comparative study of efficient photoresists' performance

**DOI:** 10.1038/srep09235

**Published:** 2015-03-18

**Authors:** Nassir Mojarad, Jens Gobrecht, Yasin Ekinci

**Affiliations:** 1 Laboratory for Micro- and Nanotechnology, Paul Scherrer Institute, 5232 Villigen, Switzerland

## Abstract

Extreme ultraviolet (EUV) lithography at 13.5 nm is the main candidate for patterning integrated circuits and reaching sub-10-nm resolution within the next decade. Should photon-based lithography still be used for patterning smaller feature sizes, beyond EUV (BEUV) lithography at 6.x nm wavelength is an option that could potentially meet the rigid demands of the semiconductor industry. We demonstrate simultaneous characterization of the resolution, line-edge roughness, and sensitivity of distinct photoresists at BEUV and compare their properties when exposed to EUV under the same conditions. By using interference lithography at these wavelengths, we show the possibility for patterning beyond 22 nm resolution and characterize the impact of using higher energy photons on the line-edge roughness and exposure latitude. We observe high sensitivity of the photoresist performance on its chemical content and compare their overall performance using the Z-parameter criterion. Interestingly, inorganic photoresists have much better performance at BEUV, while organic chemically-amplified photoresists would need serious adaptations for being used at such wavelength. Our results have immediate implications for deeper understanding of the radiation chemistry of novel photoresists at the EUV and soft X-ray spectra.

Moore's law indicates that the number of transistors in integrated circuits should double about every two years and it has been the paradigm of the semiconductor industry for about five decades. Downscaling feature sizes allows for faster processing with more power efficiency at a lower cost^1^,^2^, but physical and practical barriers hinder this progress as they have reached nanometer-size dimensions^3^. Photolithography has been the only method that meets large-scale patterning throughput for the semiconductor industry but comes with its intrinsic limitations. In optical projection lithography systems the resolution is limited by diffraction of light, and therefore, the wavelength used for the semiconductor industry has over time experienced many transitions from visible to deep ultraviolet (DUV) of λ = 193 nm, which is currently the dominant patterning wavelength. As a continuation of this trend, extreme ultraviolet (EUV) wavelength of λ = 13.5 nm (92 eV) is the major candidate for the next-generation lithography. In the last decade, significant investment has been made in the development of EUV lithography (EUVL) for mass production of integrated circuits. Several demo and pre-production EUVL scanners have been successfully installed and the third generation is expected to meet high-volume chip manufacturing for the technology nodes with below 22 nm half-pitch (HP)^4^. In less than a decade EUVL is expected to be optimized to its best for sub-10-nm resolution manufacturing, before the patterning resolution also reaches the limit set by light diffraction. The question of how far can photolithography be used for patterning integrated circuits was raised when immersion lithography at DUV was reaching its limits and even then there were serious competitors for EUVL such as electron beam (e-beam), directed self assembly, and nanoimprint lithography^5^. The possibility of the semiconductor industry still using photolithography after EUVL reaches its resolution limits highly relies on the successful development of the relevant technologies such as optics-related elements and photoresist materials. Functional photoresists should provide the RLS characteristics, that is, resolution (R), line-edge roughness (LER), and sensitivity (S). A major issue to set for post-EUV photolithography is the wavelength of choice. Using λ ≈ 1 nm drew much attention more than two decades ago but due to various practical, physical, and chemical barriers failed to be used for sub-100 nm patterning^6^. Using λ = 6.x nm, coined as Beyond EUV (BEUV), is the most prominent candidate for patterning even smaller features using photons^1^,^2^. This specific wavelength range is proposed by industry because it could potentially meet requirements of the light source, as well as reflective and imaging optics^7^. The leap from DUV to EUV is a huge technological challenge due to several reasons such as all-in-vacuum operation, reflective projection optics, effective light-source, and material response of photoresists at substantially different photon energies. Nevertheless, once EUV technology is adapted for high-volume manufacturing, the transition from EUV to BEUV will be relatively straightforward. From the photoresist point-of-view, the radiation chemistry reveals similar properties at the two wavelengths, although the optical properties of materials rapidly change in the EUV and soft X-ray regime, due to the presence of sharp atomic absorption edges^8^.

In recent years, extensive research has been devoted to developing efficient BEUV instrumentation, mainly light sources^9^ and reflective optics^10^,^11^,^12^,^13^, demonstrating the feasibility of BEUV lithography. From the photoresist side, also, the response to BEUV illumination has been a subject of recent studies. Oyama et al. made pioneering studies on sensitivities of several photoresists at EUV, BEUV, and shorter wavelengths^14^,^15^. In addition, Anderson et al. investigated the sensitivity of high-resolution photoresists and concluded that the relative sensitivities at the two wavelengths are due to their different optical absorption coefficients^16^. In order to consider BEUV lithography as an option for future lithography generations, it is also important to explore its patterning capabilities and demonstrate that providing photoresists satisfying stringent RLS requirements are feasible. Since lithography at BEUV is in its emergence phase, there is yet no effective lithography system integrated with all the needed components such as optics, light source, and masks. Interference lithography (IL) with diffraction gratings, on the other hand, enables a platform for evaluating patterning capabilities of photoresists at a broad spectral range because it provides a wavelength-independent aerial image^17^,^18^. As illustrated in Fig. 1a, to make line-space patterns, the collimated beam illuminates a mask holding a set of two parallel gratings with periodicity *P*_g_. The 1^st^-order diffracted beams overlap at a certain distance from the mask to form a periodic aerial image. By placing a photoresist-coated wafer there, a periodic line-space pattern is obtained with HP = *P*_g_/4. Moreover, thanks to the wavelength tunability of synchrotron sources and availability of high-resolution broadband masks^19^, efficient patterning and direct comparison of photoresists at different wavelengths becomes possible in a straightforward manner^20^. Here, we report on exploring BEUV lithography at λ = 6.5 nm by patterning various photoresists with different backbone chemistries at cutting-edge resolutions, evaluating their RLS parameters, and studying some lithography limitations and the involved processes. The lithography trade-off principle indicates that within a certain backbone chemistry, reducing one or two of the RLS components comes with the cost of losing at least one of the others^21^. Therefore, studying RLS variations in these intrinsically different resists paves the way for understanding the appropriate chemistry for effective BEUV lithography.

## Results

We tested the lithographic performance of three high-performance photoresists, at EUV and BEUV. Inpria XE15IB (IB) is a modern hafnium-based inorganic photoresist^22^, which has excellent sensitivity and has been demonstrated to be used for photolithography at highest resolutions^23^,^24^. The second photoresist we study is hydrogen silsesquioxane (HSQ), an inorganic photoresist with very high resolution but low sensitivity. It has been extensively used for e-beam and EUV lithography^25^, and its well-known chemistry makes it a good candidate for analyzing its response to high-energy photons and electrons. The third photoresist we study is an organic chemically amplified resist (CAR), which is among the highest resolution resists of this kind, and has an intermediate dose.

**Figure 1:**
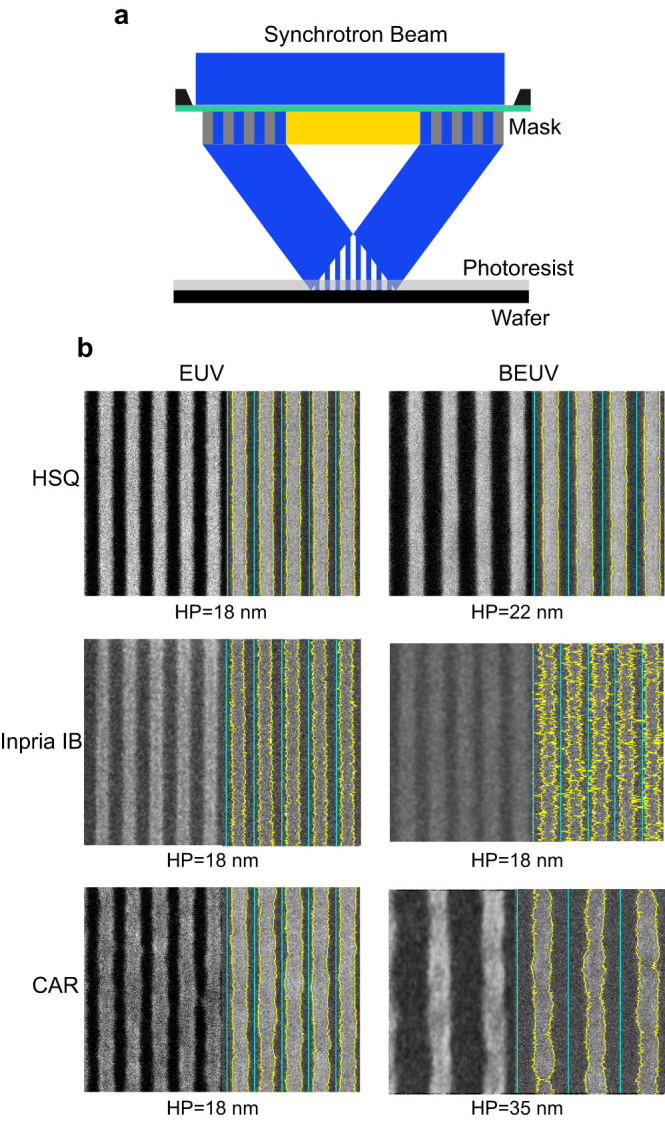
Interference lithography and patterning line-space structures. (a) Schematic of interference lithography using a broadband mask for making line-space patterns. (b) Scanning-electron micrographs of Inpria IB (first row), HSQ (second row), and CAR (third row) at EUV (left column) and BEUV (right column). The half-pitch of each image is HP_min_ and is stated under it. Yellow traces show the analyzed line profile, overlaid on the corresponding lines.

We examine patterning properties at the two wavelengths by using a broadband IL mask^19^ containing gratings with various periodicities, down to *P*_g_ = 72 nm, corresponding to HP = 18 nm on wafer. Details of mask fabrication are described in Methods. For each wavelength, the gap between the mask and the wafer is adjusted to result in maximum overlap of the diffracted beams (Fig. 1a) and a dose scan is performed on the same wafer. We note that in IL, depth-of-focus is not an issue since the aerial image is independent of the gap. Thus, our tool provides the same aerial image at different wavelengths and enables using the same mask and wafer at different wavelengths and therefore makes it possible to compare the patterning capabilities of photoresists at different wavelengths in a simple manner. Figure 1b illustrates scanning-electron micrographs (SEMs) of the three photoresists patterned at both wavelengths with their minimum patternable feature size (HP_min_), where lines were distinctly separated throughout the patterned area and revealed no bridging or pinching. As it can be seen, the three photoresists have been patterned at EUV down to HP = 18 nm, while only the Inpria resist could provide such resolution at BEUV. HSQ patterns have very smooth edges at both wavelengths but the LER increases in the other two photoresists. Moreover, the fitted traces on the Inpria resist line edges clearly indicate that for this photoresist, patterning at BEUV noticeably increases the LER and reduces the patterning contrast. In the case of the CAR, the achieved HP_min_ is almost two times higher than that at EUV and the lines reveal clear necking. We point out that HP_min_ = 18 nm is the smallest feature sized achievable with this mask and experiments are planned for determining more accurate resolution limits by making masks hosting gratings with smaller pitches.

**Figure 2:**
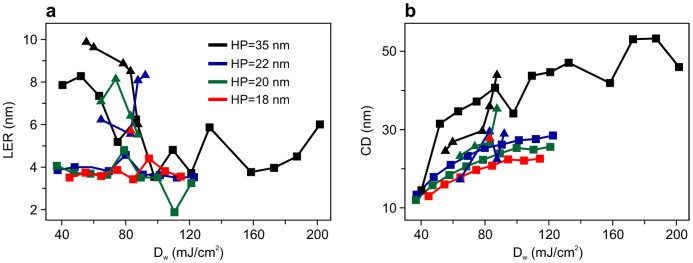
Line-edge roughness and critical dimension of Inpria IB. (a) LER and (b) critical dimension as a function of dose-on-wafer for different half-pitch values, exposed at EUV (squares) and BEUV (triangles).

**Figure 3:**
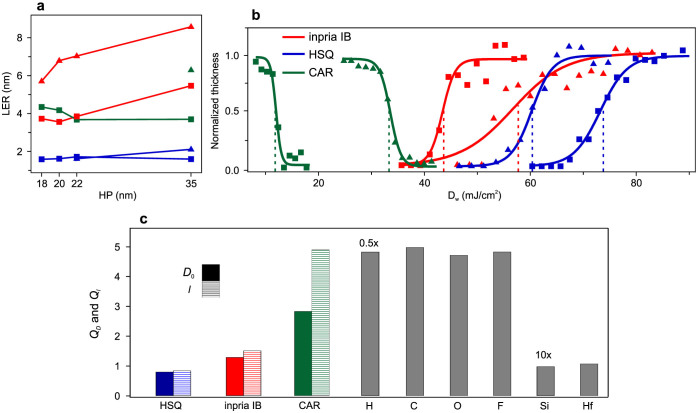
Resolution, line-edge roughness and sensitivity characterization of the three photoresists. (a) LER as a function of HP of Inpria IB (red), HSQ (blue), and CAR (green), exposed at EUV (squares) and BEUV (triangles). (b) Normalized thickness of the photoresists exposed through an open frame as a function of *D*_w_. Dashed line indicate *D*_0_ values for each case. (c) Measured *Q*_D_ (solid) and calculated *Q_l_* (dashed) values of the photoresists. Gray bars represent calculated *Q_l_* values for elements present in the chemical formula of the photoresists.

The critical dimension (CD) and LER dose-dependence is illustrated in Fig. 2. The analysis of the CD and LER was done using a commercial software (SuMMIT®) and LER values correspond to 3σ deviation averaged over both walls of 20 lines. The horizontal axis has been calibrated to represent dose-on-wafer, *D*_w_ and not the commonly used dose-on-mask *D*_m_. This calibration allows comparison of the exact dose at the wafer and takes into account the mask diffraction efficiency difference at the two wavelengths. Details of the procedure are described in Methods. As it can be seen, since Inpria IB is a negative-tone resist, for all HPs CD monotonically increases by increasing *D*_w_, and remarkably, the exposure latitude (EL) drops at all HPs when moving from EUV to BEUV. The ratio of the ELs at BEUV to EUV, *Q*_EL_ is 0.25 for HP = 35 nm and increases to *Q*_EL_ ≈ 0.4 for HP = 20 and 22 nm, yet at the HP = 18 nm illuminated by BEUV, only patterns could be formed at one dose value. Within the same functional dose range, the dependence of the LER on *D*_w_ is illustrated in Fig. 2b. Clearly, the LER is lower at all HP values when exposed with EUV in comparison to BEUV. Whether rougher edges are solely because of the chemical formulation of the resist, or because of an intrinsic property of exposure at higher photon energies can be examined by comparing patterning performance of other photoresists. Figure 3a demonstrates the average LER as a function of HP for all photoresists. In terms of LER, HSQ has a good performance at both wavelengths, and provides LER < 2 nm, whereas for Inpria IB and the CAR, patterning at BEUV results in rougher edges. For HSQ, BEUV exposures provided HP_min_ = 22 nm, while with EUV HP_min_ = 18 nm was achieved, which confirms similar measurements that show HP_min_ ≈ 15 nm^26^. In principle this resolution limit for HSQ might be improved by using another developer based on NaOH solution, commonly used for developing patterns with HP < 15 nm^24^,^26^. However, this improvement comes with the cost of losing sensitivity by a factor of 3 at EUV wavelength. The CAR we studied is among the highest-resolution resists and revealed down to HP_min_ = 18 nm at EUV. However, the smallest feature size observed at BEUV was only HP_min_ = 35 nm. In a previous study we demonstrated that another CAR, which has the EUV resolution limit of HP_min_ = 22 nm, can also be patterned with the same resolution at BEUV^20^. It could therefore be concluded that 35 nm is not the patterning resolution limit of organic CARs and that their performance is strongly dependent on their chemical composition and the incident photon energy.

The photoresist sensitivity is the next parameter that was addressed by making a dose-to-clear exposure. In this process a thin layer of spin-coated photoresist (~30 nm) is exposed through an open frame at different dose values and after development the photoresist height is mapped as a function of *D*_w_. The 50% clearance of the fitted function, *D*_0_, is regarded as the photoresist sensitivity. Although it is suggested that the absorbed dose per unit mass is a more general quantity for characterizing the sensitivity^15^, the above defined *D*_0_ has also proven to be an accurate measure of the sensitivity in other similar studies^16^, without requiring the chemical and physical properties of the resists, and is thickness independent for our very thin (<50 nm) layers. Figure 3b illustrates the normalized contrast curves of the three resists at the two wavelengths. By comparing the sensitivities at the two wavelengths (Table 1), it can be seen that HSQ is the only photoresist that requires less dose at BEUV than at EUV. Interestingly, for Inpria IB the sensitivities are quite comparable while for the CAR the BEUV sensitivity is noticeably worse. From the same curves, the photoresist contrast, γ, defined as γ = 1/log_10_(*D*_2_/*D*_1_) is also extracted and listed in Table 1. In this relation *D*_1_ is the highest/lowest dose and *D*_2_ is the lowest/highest dose at which a positive/negative-tone resist is 100% dissolved/remained after exposure and development. The γ quantity is conventionally used to evaluate different aspects of a resist performance. Going from EUV to BEUV, γ values significantly decrease for Inpria IB, and increases for HSQ and the CAR.

## Discussion

In common photoresists, where the latent image formation is governed by processes such as molecular bond scission or crosslinking, the sensitivity of the resist is proportional to its optical absorption coefficient, and as a result it is expected that *D*_0_∝*l*, where *l* is the resist attenuation length. We define *Q_D_* as the ratio of the measured *D*_0_ at BEUV to EUV, and similarly *Q_l_* as the ratio of the theoretically calculated *l* at the two wavelengths. Figure 3c illustrates *Q_D_* (solid bars) and *Q_l_* (dashed bars) for the photoresists, as well as *Q_l_* values for the elements that compose them. The *l* values were calculated based on the chemical formulations using reference^27^ and in the case of Inpria IB and the CAR, although the chemical formula is not disclosed, the companies provided us with the optical constants at the two wavelengths and *l* was deduced accordingly. As mentioned earlier, HSQ has higher sensitivity at BEUV, which is attributed to the very high absorption of Si in its backbone chemistry^14^. An interesting feature of HSQ is that *Q_D_* ≈ *Q_l_*, which indicates that the cross-linking rate of the matrix mainly depends on the absorption of its atoms. For a certain photoresist the amount of dose-to-clear depends on the number of absorbed photons as well as their energy. When a high-energy photon strikes a photoresist matrix, it loses energy mainly by creating secondary electrons (SEs), and these SEs scatter in the resist and further produce lower energy SEs until the SE energies are below the ionization energy. When comparing the SE generation using EUV and BEUV, the later has a higher energy and consequently produces a larger number of SEs per absorbed photon. On the other hand EUV photons have lower energy and as a result, for a certain amount of dose more photons exist to start the SE generation process. According to Fig. 3c, this balance results in *D*_0_∝*l* for HSQ and Inpria IB, while for the CAR the required dose at BEUV is significantly less than what is expected if the dynamics were solely governed by the absorption of the host matrix. Therefore, within the energy range that we study, exposing CARs using higher energy photons increases the chemical amplification effect, and reduces the required dose.

The effect of the resist contrast also plays a noticeable role in the final pattern quality. Quantitative comparison of this effect can be made by considering the γ values (Table 1), where larger γs represent higher contrast in the resists and consequently higher pattern quality. In the case of HSQ, these values are very similar and in fact slightly larger for BEUV than EUV. In the case of Inpria IB, γ is noticeably less at the higher photon energy, which directly reveals itself in the worse patterning contrast, that can be seen in Fig. 1b. In the case of the CAR, although γ is larger for the BEUV exposure, the dynamics of the interaction of higher energy photons and the generated SEs with the resist, together with the acid diffusion process does not allow high resolution patterning at the level that is possible at EUV. From the technological viewpoint, an important issue to address is the limitations that are held for the chemical compositions of BEUV resists. Most present low-dose EUV resists, including our studied CAR, owe their sensitivity to chemical amplification and the high photon absorption of their organic backbone chemistry. Indeed C, O, and F are common elements of many efficient EUV photoresists, and as it can be seen in Fig. 3c, their absorption is about 5 times less at BEUV. As a result, the issue of sensitivity will be a barrier for BEUV lithography of organic photoresists. On the other hand, after a rapid growth in developing organic CARs, in recent years there has been a raising trend to overcome its limitations using inorganic photoresists^22^,^28^,^29^, which could be a promise for their implementation also at BEUV.

Among the photoresist we studied Inpria IB is the only one that could be successfully patterned at BEUV with HP < 22 nm. Beside this high resolution it provides, the LER is noticeably higher in comparison to EUV patterning. To find the reason for this higher LER, we take a further step of analyzing it into its main components. LER sources could be divided into three main categories, namely that associated with shot noise (LER_SN_), the photoresist material (LER_mat_), and the process (LER_proc_)^30^, that add up quadratically such that the total LER can be written as follows:
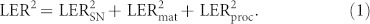
Shot noise is caused by the random fluctuations of the limited number of absorbed photons, which follows Poisson statistics, and reveals itself in the patterned lines as variations at the edges of the exposed structures. Assuming that the resist absorbs a dose of *D*_a_, corresponding to *N*_a_ photons at wavelength λ, it is commonly known that LER_SN_∝1/√*N*_a_^31^,^32^. Moreover, since *N*_a_ = *D*_a_ λ/*hc*, where *h* and *c* are accordingly the Planck's constant and the speed of light in vacuum, it could be derived that:
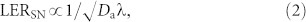
which implies that LER_SN_ increases at shorter wavelengths. In a hypothetical resist which has identical *D*_a_ at both EUV and BEUV, *Q*_LER_ = 1/√ *Q*_λ_ = 1.44, where *Q*_LER_ and *Q*_λ_ are respectively the ratios of the LER and λ at BEUV to EUV. Although real resists could not be treated in that way, we can still investigate whether shot noise alone can justify the large value of *Q*_LER_ in Inpria or not. According to Fig. 3a, for HP = 22 nm we experimentally measure Q_LER_ = 1.83, while Fig. 3c indicates *Q_D_* = 1.31 and consequently, according to Eq. 2, *Q*_LER_ only caused by shot noise is 1/√ *Q*_D_ *Q*_λ_ = 1.26. This means that the pure shot noise cannot justify the discrepancy of LER at the two wavelengths, and LER_mat_ and LER_proc_ also have a noticeable effect. Among the process factors, what significantly changes the patterning quality is flare cause by defects on the masks and grating roughness. There is no direct study of the effect of grating roughness on the final aerial image in an IL layout. However, based on the fact that the induced speckles cause by mask roughness directly influence the LER_proc_^33^, reducing the wavelength increases the LER_proc_. Regarding LER_mat_, for a certain photoresist most material factors remain the same for the two wavelengths. The main difference is the number of generated SEs and their energy distribution and diffusion into the matrix that depends on the incident photon energy. Quantitative derivation of this dependence can be simulated using a cellular automaton model^31^, which is beyond the scope of this article and is subject to further investigations. One way to lower LER_mat_ is to use alternative development chemistries, but this often comes with the cost of losing sensitivity.

**Figure 4:**
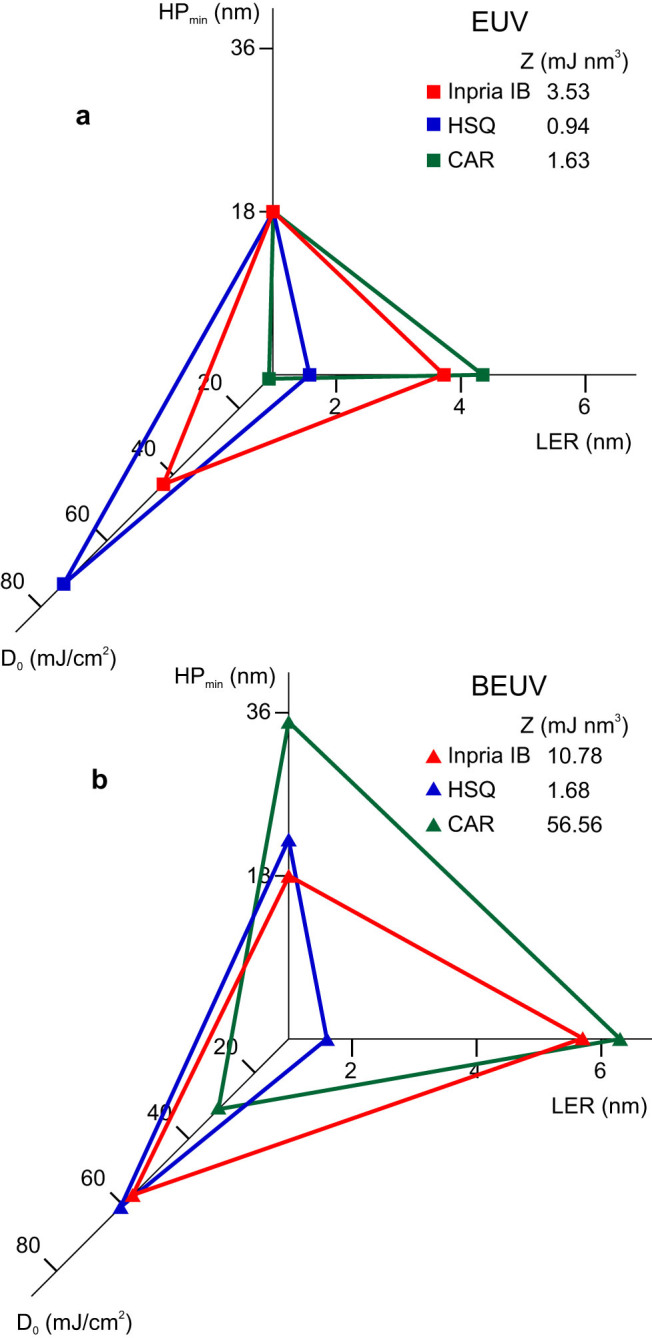
Z-parameters. Overall RLS characterization of the photoresists and their evaluated Z-parameters. At (a) EUV and (b) BEUV.

Quantitative evaluation and comparison of the performance of different resists and their dependence on λ can be made by a figure of merit. One such rigorous measure is the so-called *K*_LUP_^21^, which has been a standard parameter for assessing the performance of CARs that meet the semiconductor industry's specifications. *K*_LUP_ is derived explicitly from the relations between each RLS element with other involved microscopic quantities, including the incident photon energy^21^. The limitation of using such quantity in our evaluation is that that *K*_LUP_ can only be used for chemically amplified resists, which have a well defined acid diffusion length. Therefore, in this study we use the so-called *Z-parameter* for this purpose, since it reveals the explicit relation between macroscopic RLS parameters, is not limited to chemically amplified resists, and has been shown to be an effective and accurate tool for resist evaluation^34^,^35^. The *Z*-parameter adapted to our conventions reads as follows:
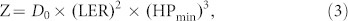
where LER corresponds to that of the smallest patterned features with HP_min_. Figure 4 illustrates the RLS triangle for all the studied cases. The obtained values at both wavelengths are within the same order of magnitude of other important EUV resists^23^,^34^. It should be noted that these Z-parameters were evaluated using an IL mask that allowed the printing of line-space structures down to HP = 18 nm and more compact features were not patterned due to nanofabrication complications that rapidly increase at shorter *P*_g_s. Therefore HP_min_ for Inpria IB and HSQ was taken as far as we were able to get using this mask. HP_min_ < 10 nm features have been reported for EUV exposures on Inpria IB and HSQ^23^,^24^ and therefore finding the exact resolution limits at BEUV requires efficient masks with higher resolution. The very comparable Z-parameter at the two wavelengths for HSQ clearly demonstrates the key role of highly absorbing Si and suggest using similar elements, such as phosphor and sulfur, for the development of specific BEUV photoresists. As for HSQ itself, the barrier for large-scale utilization of such a resist at BEUV is its worse resolution performance in comparison to EUV, as well as its low sensitivity. It seems that a resist such as Inpria IB would be the best candidate among what has been studied because of a reasonably low Z-parameter at both wavelengths as well as the possibility to provide < 22 nm resolutions for future technology nodes. For Inpria IB, the main cause of the Z-parameter difference at the two wavelengths is the difference in the LERs, which as explained earlier, can be improved by the reduction of LER_proc_ using higher quality mask fabrication. When it comes to CAR patterning, the Z-parameter reveals a noticeable difference between the resist performance at the two wavelengths. This is caused by the worse performance of all the three RLS parameters at BEUV, mainly due to the CAR's organic composition, and the acid diffusion process, which amplifies the lower quality of the lines after exposure and development.

In conclusion, lithography at EUV is still the main candidate of high-volume chip manufacturing for the semiconductor industry. Should EUVL reach its single-digit patterning resolution, the choice of using 6.x nm photons for further advancing the resolution requires investments in all aspects of its technology, including light source, optics, and photoresist. Moving to shorter wavelengths for lithography faces the intrinsic increase of LER, caused by shot noise. Moreover, the aerial image quality is more sensitive to defects and roughness in the optics, which also raises the LER. The capability of BEUV photoresist matching the RLS requirements highly depends on their chemical composition. Organic photoresists have very low absorption at BEUV and are unlikely to satisfy sensitivity standards. Using organic CARs to lower the required dose, as we have shown, results in compensation for resolution and LER. It seems that inorganic photoresist using efficiently absorbing elements would be a good choice for reaching future technology nodes of the semiconductor industry with suitable sensitivity and LER. We hope that our findings could pave the way for better understanding and design of novel photoresists that are efficient and functional at photon energy ranges of EUV and beyond.

## Methods

### Photoresist parameters

All resists were spin-coated on a 4-inch p-doped Si wafer and were processed as follows: HSQ (Dow Corning® XR-1541) was spin-coated with 5000 rpm for 45 s, which results in resist thickness of 35 nm. After exposure the sample was developed in Tetramethylammonium hydroxide (TMAH) 25% for 60 s. Inpria (XE15IB) was spin-coated on a wafer treated by oxygen plasma, with 2500 rpm for 45 s, which provides a 20-nm-thick resist layer. The wafer was then post-apply baked (PAB) at 80°C for 120 s and after exposure post-exposure bake (PEB) with the same temperature and time of PAB. The development is also in TMAH 25% for 120 s. For CAR, an adhesion underlayer was first prepared and on top, the photoresist was spin-coated with 2500 rpm for 60 s, which provided a 30-nm-thick layer, and was followed by PAB of 130°C for 60 s. PEB was at 110°C for 60 s and the development was in TMAH 0.26 N for 30 s. The spin-coating of all resists provides a smooth and uniform thin layer and after exposure and development resist thickness loss of 10%, 15%, and 10% is measured for HSQ, Inpria IB, and the CAR, respectively.

### Dose-on-wafer calibration

The *D*_w_ calibration was necessary to take into account the effect of the wavelength-dependent diffraction efficiency, η, of the mask. Experimental evaluation of η at each wavelength was done through the process described in reference^19^ by finding the ratio of *D*_0_ of the first order diffracted beam to the open frame exposure. Since at the patterned area the beam is the sum of the two diffracted beams (Fig. 1a), *D*_w_ is given as: *D*_w_ = 2η *D*_m_.

### The broadband IL mask

The masks have the benefit of efficiently diffracting light at both EUV and BEUV wavelengths as well as blocking the non-diffracted beams. A broadband mask consists of HSQ line-space gratings and an Au photon stop, made on a free-standing 100 nm thick Si_3_N_4_ membrane. On each mask HSQ gratings with different periodicities, in the range of 72 < *P*_g_ < 200 nm and the height of ~100 nm, are patterned by e-beam lithography. An 8 nm thick Cr layer is evaporated on Si_3_N_4_ membrane, prior to spin-coating HSQ, and after fabricating the gratings the Cr between the grating lines are removed by reactive-ion etching to increase the diffraction efficiency. This Cr layer serves mainly as a conductive layer during the e-beam lithography step to reduce the sub-field stitching effect. The Au photonstop is ~600 nm thick and covers the area on the membrane not holding the HSQ gratings. Theoretical calculations and experimental evaluations show that such grating configuration has an IL efficiency of ~8% at EUV and ~2% at BEUV. Further Details on the fabrication steps can be found in reference^19^.

## Author Contributions

N.M. and Y.E. conceived the experiments. N.M. carried out the mask fabrication, performed the experiments and characterizations, and analyzed the data. Y.E. and J.G. provided expertise on the nanofabrication. N.M. and Y.E. wrote the manuscript. All authors discussed the results and contributed to the final version of the manuscript.

## Figures and Tables

**Table 1:** *D*_0_ and γ values of the three photoresists at EUV and BEUV wavelengths

	*D*_0_ (mJ/cm^2^)		γ	
	EUV	BEUV	EUV	BEUV
**HSQ**	73.8	61.0	11.76	11.79
**Inpria IB**	43.3	56.7	14.08	4.84
**CAR**	11.6	33.2	7.49	11.17
